# TT-10–loaded nanoparticles promote cardiomyocyte proliferation and cardiac repair in a mouse model of myocardial infarction

**DOI:** 10.1172/jci.insight.151987

**Published:** 2021-10-22

**Authors:** Wangping Chen, Danielle Pretorius, Yang Zhou, Yuji Nakada, Jinfu Yang, Jianyi Zhang

**Affiliations:** 1 Department of Biomedical Engineering, School of Medicine and School of Engineering, University of Alabama at Birmingham, Alabama, Birmingham, USA.; 2 Department of Cardiovascular Surgery, Second Xiangya Hospital, Central South University, Changsha, China.; 3 Department of Medicine and Cardiovascular Diseases, University of Alabama at Birmingham, Birmingham, Alabama.

**Keywords:** Cardiology, Therapeutics, Cell cycle, Drug therapy, Nanotechnology

## Abstract

The meager regenerative capacity of adult mammalian hearts appears to be driven by the proliferation of endogenous cardiomyocytes; thus, strategies targeting mechanisms of cardiomyocyte cell cycle regulation, such as the Hippo/Yes-associated protein (Hippo/Yap) pathway, could lead to the development of promising therapies for heart disease. The pharmacological product TT-10 increases cardiomyocyte proliferation by upregulating nuclear Yap levels. When intraperitoneal injections of TT-10 were administered to infarcted mouse hearts, the treatment promoted cardiomyocyte proliferation and was associated with declines in infarct size 1 week after administration, but cardiac function worsened at later time points. Here, we investigated whether encapsulating TT-10 into poly-lactic-co-glycolic acid nanoparticles (NPs) before administration could extend the duration of TT-10 delivery and improve the potency of TT-10 for myocardial repair. TT-10 was released from the TT-10–loaded NPs for up to 4 weeks in vitro, and intramyocardial injections of TT-10–delivered NPs stably improved cardiac function from week 1 to week 4 after administration to infarcted mouse hearts. TT-10–delivered NP treatment was also associated with significantly smaller infarcts at week 4, with increases in cardiomyocyte proliferation and nuclear Yap abundance and with declines in cardiomyocyte apoptosis. Thus, NP-mediated delivery appears to enhance both the potency and durability of TT-10 treatment for myocardial repair.

## Graphical abstract

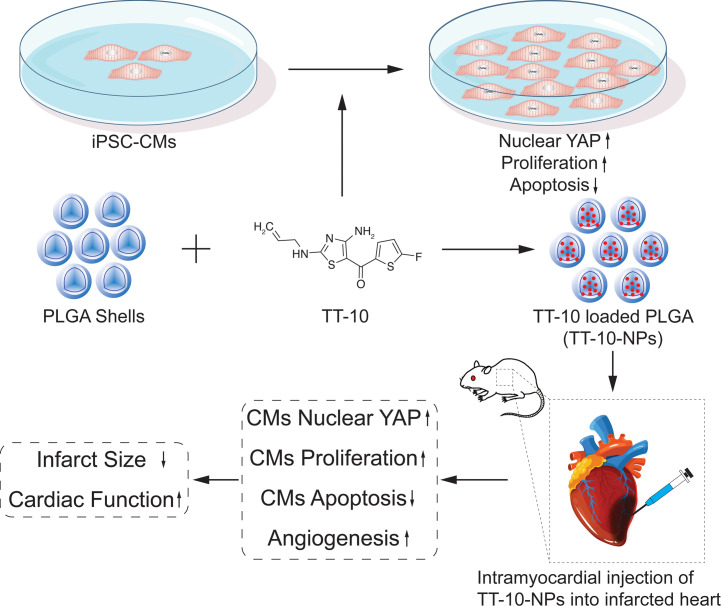

## Introduction

The endogenous regenerative capacity of adult mammalian hearts is exceptionally limited (1) and cannot replace the cardiomyocytes that are lost to myocardial infarction (MI) or other cardiac disorders. However, when apical resection surgery was performed in mice or pigs shortly after birth, and MI was experimentally induced several weeks later, the animals recovered completely, with little evidence of scarring (1, 2). Furthermore, recovery appeared to be driven primarily by the proliferation of preexisting cardiomyocytes, suggesting that postnatal cardiomyocytes retain some latent capacity for proliferation and that strategies targeting mechanisms of cardiomyocyte cell cycle regulation could lead to the development of promising new therapies for heart disease.

Hippo signaling has a key regulatory role in fetal cardiac development (3, 4), and 2 downstream components of the Hippo pathway, YES-associated protein (YAP) and transcriptional coactivator with PDZ-binding motif (TAZ), have recently been linked to cardiac regeneration (5, 6). The pharmaceutical product TT-10 (C_11_H_10_FN_3_OS_2_) is a fluorine substituent of TAZ-12, and it appears to promote cardiomyocyte proliferation: in mice, intraperitoneal injections of TT-10 reduced infarct size 1 week after MI but did not prevent progressive declines in cardiac function at later time points (7). Previously, we have shown that when growth factors or small-molecule inhibitors are loaded into poly-lactic-co-glycolic acid (PLGA) nanoparticles (NPs; ref. 8), the chemicals are slowly released from the NPs over a period of up to 4 weeks (9, 10). Thus, we investigated whether encapsulating TT-10 into PLGA NPs before administration could extend the duration of TT-10 delivery and improve the potency of TT-10 for treatment of MI.

## Results

### TT-10 promoted cell cycle activity, reduced apoptosis, and upregulated Yap signaling in cultured cardiomyocytes.

**Figure 1:**
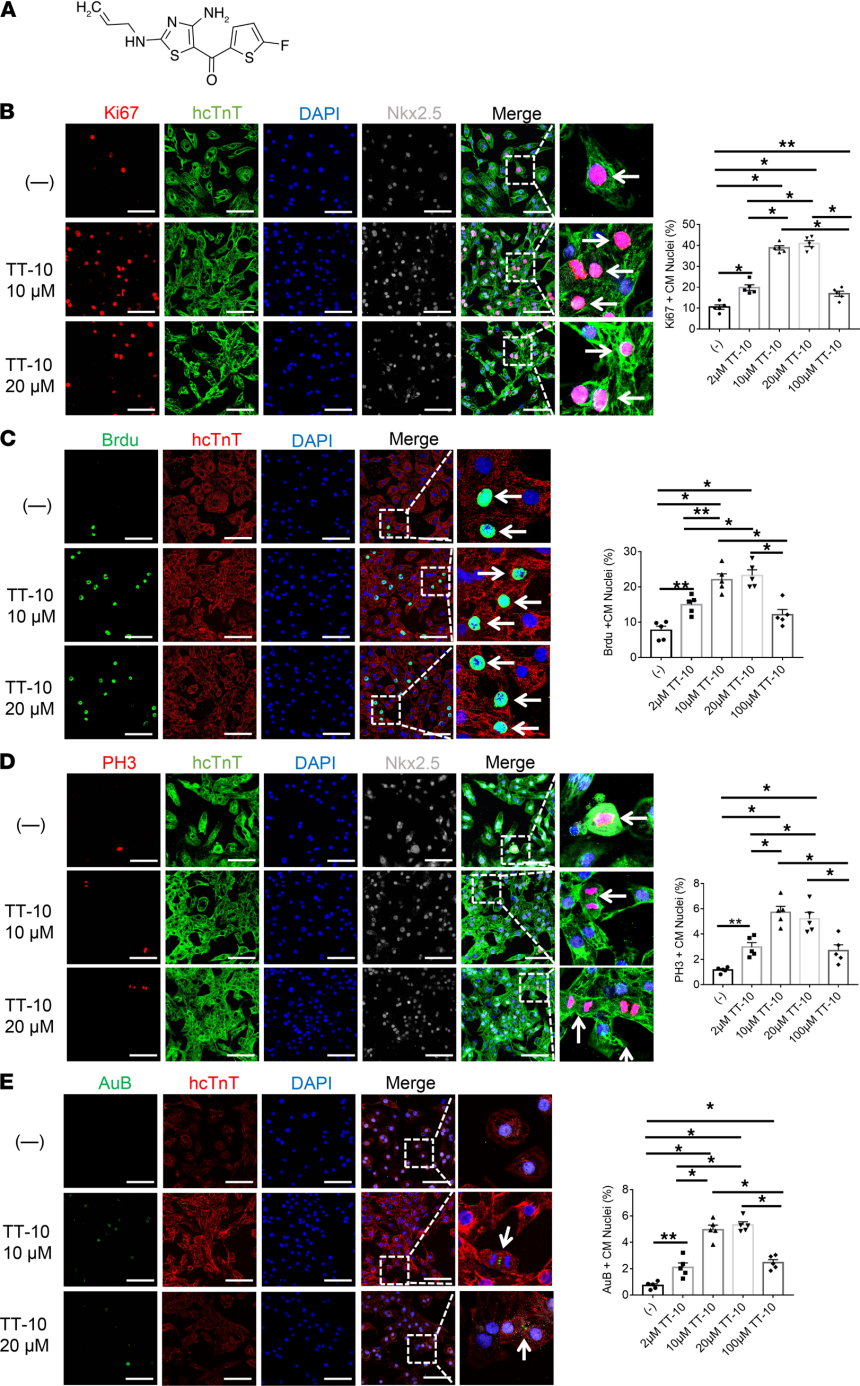
TT-10 increased cell cycle activity and proliferation in cultured hiPSC-CMs. (**A**) TT-10 (C_11_H_10_FN_3_OS_2_; molecular weight, 283.34 Da) contains 4 C=C, 1 C=N, and 1 C=O double bond. Maximum UV/Vis absorbance is approximately 200 nm. (**B**–**E**) hiPSC-CMs were cultured under standard conditions (–) or treated with varying concentrations of TT-10 (2 μM, 10 μM, 20 μM, or 100 μM) for 48 hours and immunofluorescently stained for the human isoform of cardiac troponin T (hcTnT). Nuclei were identified by staining with DAPI or with DAPI and Nkx2.5 antibodies. (**B**) Proliferation was evaluated via immunofluorescence costaining for Ki67 and quantified as the percentage of Ki67-positive cells. (**C**) hiPSC-CMs in the S phase of the cell cycle were identified via immunofluorescence costaining for BrdU incorporation and quantified as the percentage of BrdU-positive cells. (**D**) hiPSC-CMs in the M phase of the cell cycle were identified via immunofluorescence costaining for PH3 and quantified as the percentage of PH3-positive cells. (**E**) hiPSC-CMs undergoing cytokinesis were identified via immunofluorescence costaining for Aurora B (AuB) and quantified as the percentage of AuB-positive cells. Scale bar: 20 μm. All experiments were repeated 5 times. *P < 0.01, **P < 0.05, 1-way ANOVA with Tukey’s correction.

**Figure 2:**
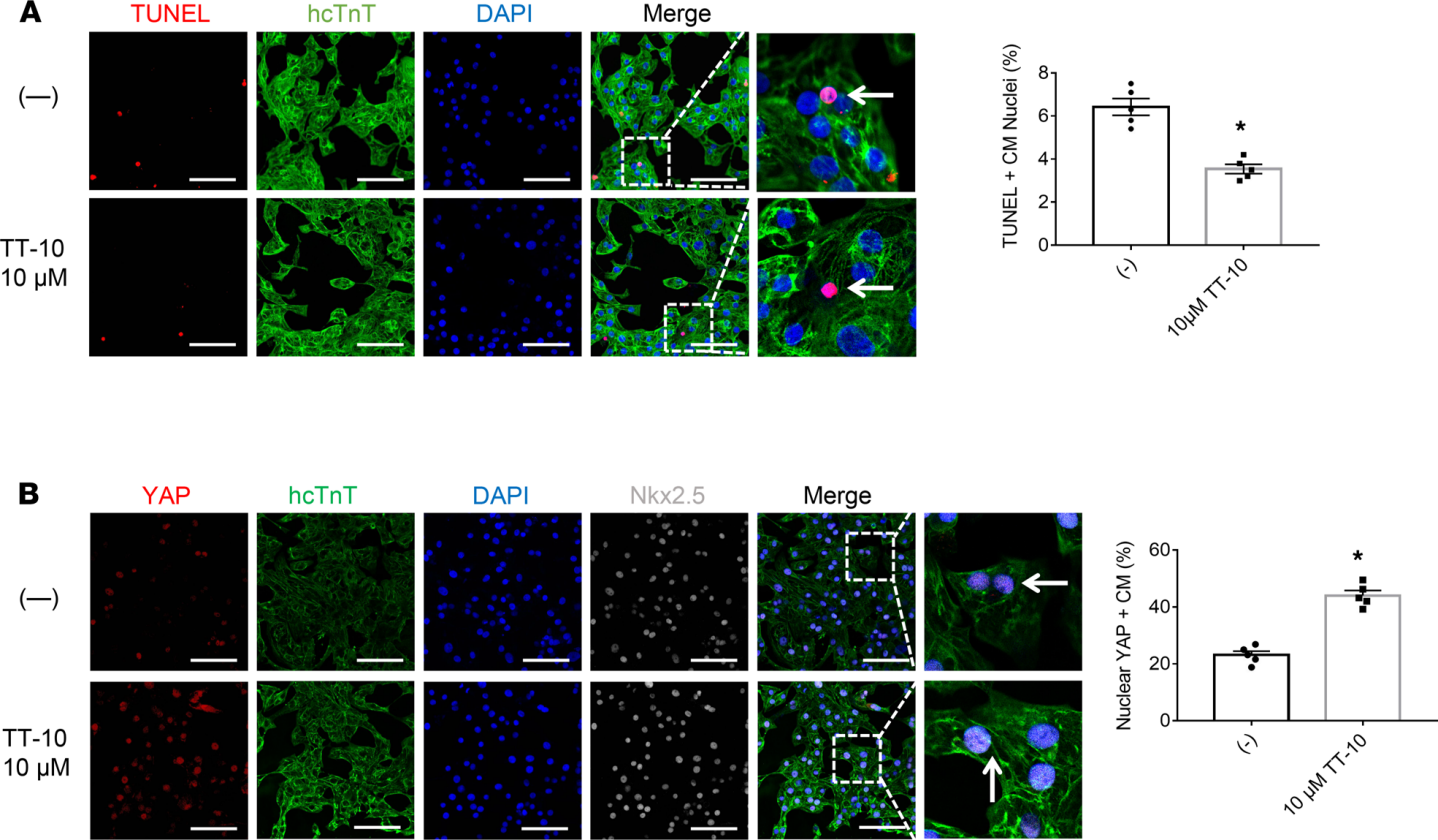
TT-10 enhanced survival and nuclear Yap levels in cultured hiPSC-CMs. (**A**) Apoptosis was evaluated via TUNEL staining and quantified as the percentage of TUNEL-positive cells. (**B**) hiPSC-CMs were immunofluorescently costained for Yap, and the proportion of cells with Yap-positive nuclei is presented as a percentage. Scale bar: 20 μm. All experiments were repeated 5 times. *P < 0.01, Student’s *t* test.

The effect of TT-10 (Figure 1A) on cardiomyocyte cell cycle activity and proliferation was determined by culturing human induced pluripotent stem cell–derived cardiomyocytes (hiPSC-CMs) in varying concentrations of TT-10 (0, 2 μM, 10 μM, 20 μM or 100 μM) for 48 hours and then evaluating the presence of markers for cell proliferation (Ki67 expression, Figure 1B), S phase (BrdU incorporation, Figure 1C), and M phase (histone 3 phosphorylation [PH3], Figure 1D) of the cell cycle and for cytokinesis (Aurora B, Figure 1E). When visualized via immunofluorescence, the proportion of cells displaying each of the 4 markers increased as the TT-10 concentration was raised from 0 to 2 μM, peaked at 10 to 20 μM TT-10, and then declined at 100 μM TT-10. TT-10 treatment (10 μM) also significantly reduced measures of hiPSC-CM apoptosis (TUNEL staining, Figure 2A) and significantly increased the proportion of hiPSC-CMs with Yap-positive nuclei (Figure 2B), which is consistent with the role of Hippo/Yap signaling in cardiac regeneration (4, 5). The effect of TT-10 on YAP pathway activity was also evaluated via Western blot (Supplemental Figure 1; supplemental material available online with this article; https://doi.org/10.1172/jci.insight.151987DS1): total Yap levels in hiPSC-CMs treated with or without TT-10 were similar, but phosphorylated Yap (p-Yap) was less abundant in TT-10–treated cells. Thus, because p-Yap is sequestered and degraded in the cytosol, while unphosphorylated Yap translocates to the nucleus where it induces the expression of genes that regulate cell proliferation and survival, these results suggest that the effect of TT-10 treatment on cell cycle progression and apoptosis in cultured cardiomyocytes was likely mediated, at least in part, by increases in Yap signaling.

### NP-mediated delivery increased the potency of TT-10 for myocardial repair in a mouse MI model.

**Figure 3:**
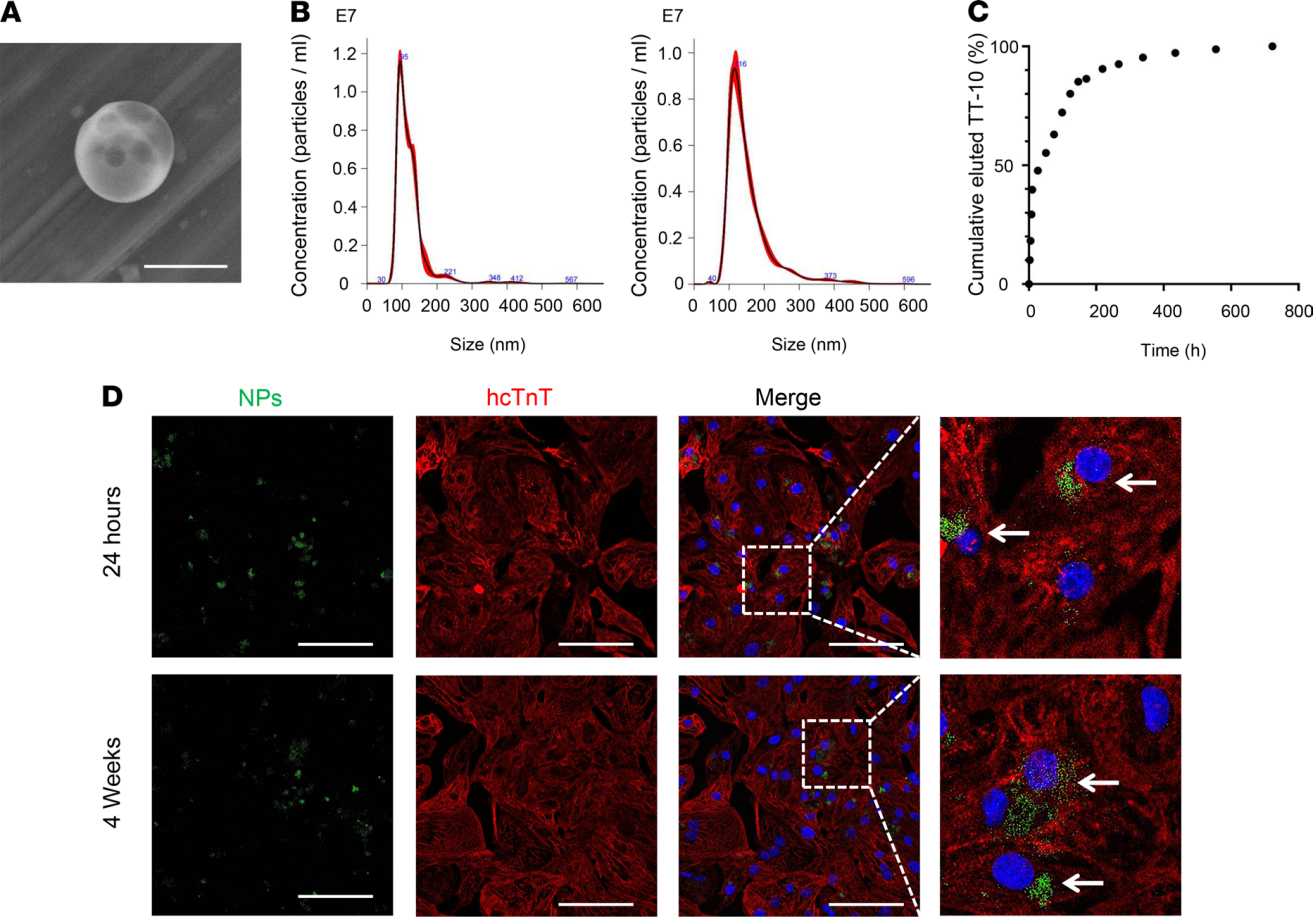
Cultured TT-10–NPs released TT-10 for more than 4 weeks and were taken up by hiPSC-CMs. (**A**) PLGA NPs were imaged via scanning electron microscopy. A representative image of a TT-10–NP is shown. Scale bar: 200 nm. (**B**) The size distribution of the empty PLGA NPs (Empty-NP) (left) and TT-10–NPs (right) was evaluated via nanoparticle tracking analysis. (**C**) The kinetics of TT-10 release from TT-10–NPs were determined with a dialysis device. Suspensions of TT-10–NPs were incubated at 37°C, and samples of the elution medium were withdrawn and replaced at the indicated time points. Then, the cumulative percentage of TT-10 that had been released from the NPs was calculated for each time point. (**D**) hiPSC-CMs were cultured with NPs (2 μg/mL) that had been loaded with coumarin-6 (green) for 24 hours or 4 weeks. Then, the cells were immunofluorescently stained for hcTnT (red), and nuclei were identified by DAPI staining (blue). Internalized NPs are identified with arrows. Scale bar: 20 μm.

MI was induced via permanent ligation of the left anterior descending (LAD) coronary artery, and then the animals were randomly assigned to treatment with Dulbecco’s PBS (DPBS), empty PLGA NPs (Empty-NPs), TT-10 solution (TT-10–SOL), or TT-10–loaded PLGA NPs (TT-10–NPs). A fifth group of animals (the sham group) underwent all surgical procedures for MI induction except arterial ligation. Before TT-10 administration, the mean diameters of the Empty- and TT-10–NPs were 123.3 ± 1.7 nm and 155.4 ± 0.7 nm, respectively (Figures 3, A and B). Loading efficiency was found to be 2.50 ± 0.08 μg of TT-10 per mg PLGA NPs. And when the TT-10–NPs (1 mg) were incubated in DPBS (1 mL), 47% of the encapsulated TT-10 was released during the first day and 90% was released by day 9 (Figure 3C). TT-10–NPs also significantly increased the expression of proliferation markers in cultured hiPSC-CMs (Supplemental Figure 2), and when hiPSC-CMs were cultured with PLGA-NPs that had been loaded with a fluorescent dye (coumarin-6), immunofluorescence images confirmed that the NPs were taken up by the cells (Figure 3D).

**Figure 4:**
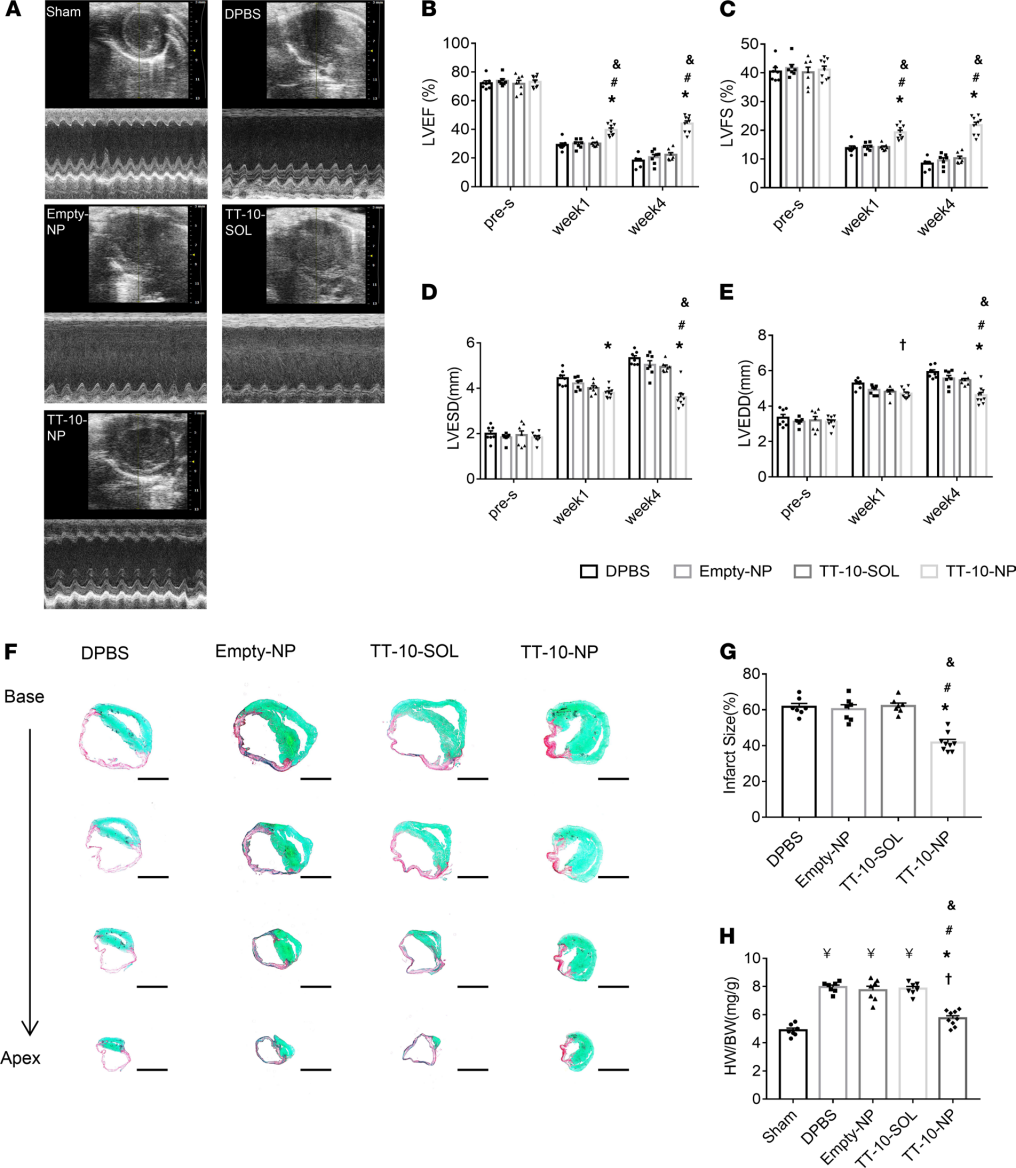
Intramyocardial injections of TT-10–NPs improved recovery from MI in mice. MI was induced in mice, and then the animals were treated with DPBS, empty PLGA NPs (Empty-NP), TT-10 solution (TT-10–SOL), or with TT-10–loaded PLGA NPs (TT-10–NP). A fifth group of animals (the sham group) underwent all surgical procedures for MI induction except arterial ligation. (**A**) Echocardiographic assessments of (**B**) left ventricular ejection fraction (LVEF), (**C**) fractional shortening (LVFS), (**D**) end-systolic diameter (LVESD), and (**E**) end-diastolic diameter (LVEDD) were conducted before MI induction (pre-s) and 1 and 4 weeks afterward. Representative images in **A** were collected at week 4. (**F**–**H**) Animals were sacrificed at week 4, and hearts were explanted. (**F**) Heart sections were stained with Picrosirius Red and Fast Green to identify regions of infarcted (red) and noninfarcted (green) tissue. Scale bar: 1 mm. (**G**) Then, infarct sizes were quantified as the ratio of the scar area to the total left ventricular surface area and expressed as a percentage. (**H**) Myocardial hypertrophy was evaluated as the ratio of the weight of the whole heart to the animal’s bodyweight (HW/BW). (**B**–**E**) *n =* 7–9 animals per group. *P < 0.01 vs. DPBS, †*P <* 0.05 vs. DPBS, ^#^*P <* 0.01 vs. Empty-NP, ^&^*P <* 0.01 vs. TT-10–SOL; 2-way ANOVA with Tukey’s multiple comparisons test. (**G** and **H**) *n =* 7–9 animals per group; ^¥^*P <* 0.01 vs. sham, †*P <* 0.05 vs. sham, *P < 0.01 vs. MI, ^#^*P <* 0.01 vs. Empty-NP, ^&^*P <* 0.01 vs. TT-10–SOL; 1-way ANOVA with Tukey’s multiple comparisons test.

Four mice died from MI induction. In the surviving animals, echocardiographic (Figure 4A) assessments of left ventricular (LV) ejection fraction (LVEF; Figure 4B), LV fractional shortening (LVFS; Figure 4C), LV end-systolic diameter (LVESD; Figure 4D), and LV end-diastolic diameter (LVEDD; Figure 4E) did not differ significantly among the DPBS, Empty-NP, and TT-10–SOL treatment groups at week 1 or week 4 after treatment; however, ejection fraction and fractional shortening were significantly greater in TT-10–NP animals at both time points, while end-systolic diameter and end-diastolic diameter were significantly lower at week 4, than in any other group that underwent MI induction. Measurements in animals treated with DPBS, Empty-NPs, or TT-10–SOL also appeared to worsen from week 1 to week 4, while measurements in the TT-10–NP group tended to remain stable (or improve slightly) over the same period. Furthermore, analyses of hearts explanted at week 4 indicated that neither the size of the infarct (Figure 4, F and G) nor the heart weight/bodyweight ratio (Figure 4H) differed significantly among the DPBS, Empty-NP, and TT-10–SOL treatment groups, but both measurements were significantly lower after treatment with TT-10–NP than in any of the other experimental treatment groups. Notably, when NPs were loaded with both TT-10 and coumarin-6 before administration to infarcted mouse hearts, immunofluorescence images of tissues from the border zone (BZ) indicated that most (~70%) of the NPs were degraded by week 1 and a small proportion (<1%) remained detectable through week 4 (Supplemental Figure 3).

### TT-10–NP administration after MI increased cardiomyocyte proliferation, reduced cardiomyocyte apoptosis, and improved vascularity in the peri-infarct region.

**Figure 5:**
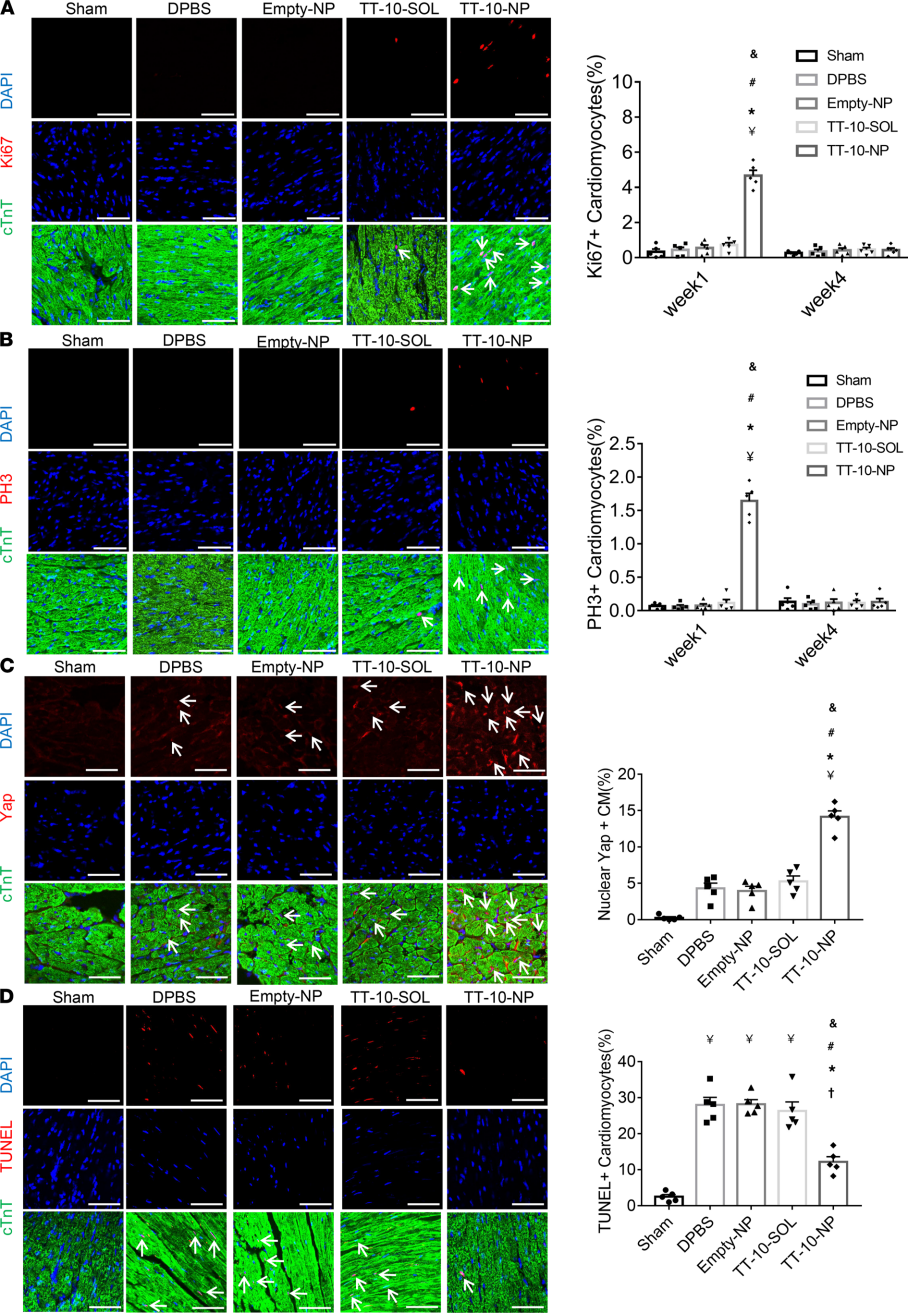
Intramyocardial injections of TT-10–NPs after MI increased cell cycle activity, proliferation, nuclear Yap abundance, and survival in cardiomyocytes. Sections from the border zones of the infarcts in animals from the DPBS, Empty-NP, TT-10–SOL, and TT-10–NP groups, and from the corresponding region of hearts from sham animals, were obtained (**A**–**C**) 1 week or (**D**) 72 hours after MI induction and treatment or sham surgery. Then, the sections were stained for cardiac troponin T (cTnT) to visualize cardiomyocytes, and nuclei were identified via DAPI staining. Scale bar: 20 μm. *n =* 5 animals per group, 4 sections per heart, and 5 high-power fields per section. (**A**) Cardiomyocyte proliferation was evaluated via immunofluorescence costaining for Ki67 and quantified as the percentage of cTnT-positive cells that also expressed Ki67. (**B**) Cardiomyocytes in the M phase of the cell cycle were identified via immunofluorescence costaining for PH3 and quantified as the percentage of cTnT-positive cells that were also positive for PH3. Representative images in **A** and **B** were obtained at week 1. (**C**) Sections were immunofluorescently costained for Yap, and the proportion of cTnT-positive cells with Yap-positive nuclei is presented as a percentage. (**D**) Cardiomyocyte apoptosis was evaluated via TUNEL staining and quantified as the percentage of cTnT-positive cells that were also positive for TUNEL. ^¥^*P <* 0.01 vs. sham, †*P <* 0.05 vs. sham, *P < 0.01 vs. DPBS, ^#^*P <* 0.01 vs. Empty-NP, ^&^*P <* 0.01 vs. TT-10–SOL; 1-way ANOVA with Tukey’s multiple comparisons test.

**Figure 6:**
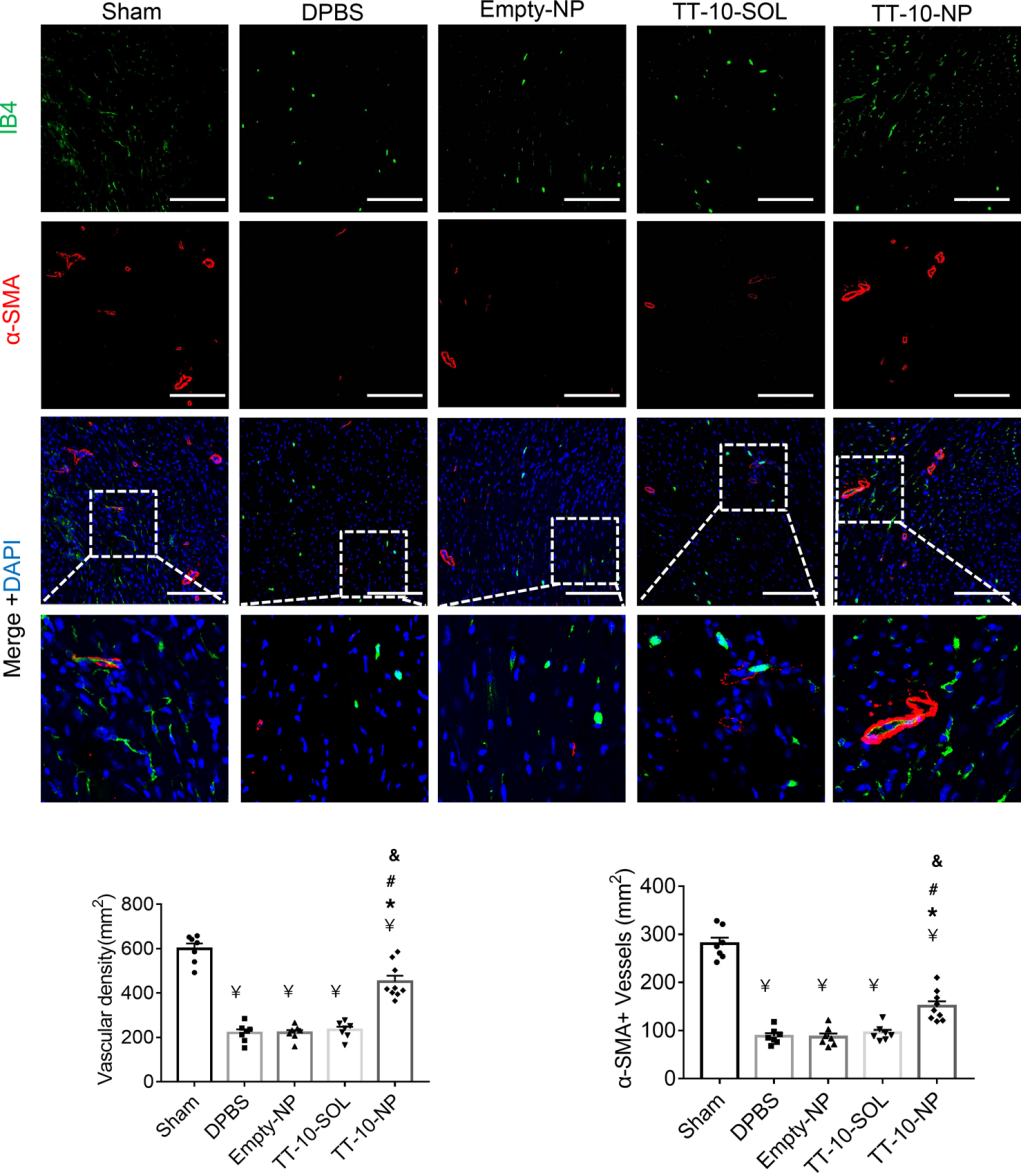
Assessment of TT-10–NP–mediated angiogenesis in the border zone of the infarct. Sections from the border zone of animals that underwent MI induction, or from the corresponding regions of hearts from sham-operated animals, were obtained at week 4 and stained with the endothelial marker isolectin B4 (IB4) and for the expression of α smooth-muscle actin (α-SMA). Nuclei were identified via DAPI staining, and then vascular density and arteriole density were quantified by determining the number of IB4-positive and α-SMA–positive vascular structures, respectively, per square millimeter. Scale bar: 50 μm. *n =* 7–9 animals per group, 4 sections per heart, and 5 high-power fields per section. ^¥^*P <* 0.01 vs. sham, *P < 0.01 vs. DPBS, ^#^*P <* 0.01 vs. Empty-NP, ^&^*P <* 0.01 vs. TT-10–SOL; 1-way ANOVA with Tukey’s multiple comparisons test.

To determine whether the improvements in myocardial recovery observed in TT-10–NP mice were at least partially attributable to the activation of Hippo/Yap signaling and cardiomyocyte proliferation, subsets of mice were sacrificed 1 week after MI induction, and then cardiomyocytes expressing Ki67 (Figure 5A), PH3 (Figure 5B), and nuclear Yap (Figure 5C) were quantified via immunofluorescence in BZ heart sections. All 3 markers were dramatically more common in BZ cardiomyocytes from TT-10–NP animals than in BZ cardiomyocytes from any other group; however, Ki67 and PH3 fluorescence were nearly undetectable in BZ sections from TT-10–NP mice sacrificed 4 weeks after injury and treatment, likely because cardiomyocytes that began actively cycling in response to TT-10–NP administration exited the cell cycle as the NPs degraded and TT-10 abundance declined. Furthermore, although apoptotic cardiomyocytes were significantly more common in BZ sections obtained from all groups at 72 hours after MI induction than in the corresponding region of hearts from sham animals, measurements were significantly lower in TT-10–NP animals than in the DPBS, Empty-NP, or TT-10–SOL treatment groups (Figure 5D). TT-10–NP administration also appeared to promote angiogenesis, because vascular structures that stained positively with the endothelial marker isolectin B4 or for the expression of α-smooth muscle actin were significantly more numerous 4 weeks after MI, and treatment in BZ sections obtained from TT-10–NP mice had the greatest number of positive vascular structures compared with those from animals in any other group that underwent MI induction (Figure 6). Thus, TT-10–NP administration appeared to improve recovery from MI by promoting angiogenesis as well as cardiomyocyte proliferation and survival.

## Discussion

The primary mechanism of cardiomyocyte renewal both for maintaining cardiac homeostasis and during recovery from myocardial injury appears to be the proliferation of preexisting cardiomyocytes (11, 12). However, mammalian cardiomyocytes undergo cell cycle arrest during the perinatal period, and the residual proliferative capacity of adult cardiomyocytes is far too low to regenerate more than a small fraction of the cardiomyocytes that are lost to MI. Thus, therapies targeting the mechanisms that regulate cell cycle activity may be among the most useful strategies for replacing the myocardial scar with functional contractile tissue, and because this approach mimics the endogenous mechanism of cardiomyocyte renewal, the newly generated cells are likely to be better integrated with the native tissue than exogenously administered cells or engineered tissues.

The Hippo signaling pathway impedes proliferation by sequestering YAP and TAZ in the cytosol. Once activated, YAP and TAZ translocate to the nucleus, where they interact primarily with transcription factors of the TEA domain (TEAD) family and the YAP/TAZ-TEAD complex induces the expression of genes that control cell proliferation and apoptosis (13). TT-10 is a fluorine substituent of TAZ-12; results from large-scale, cell-based screenings of pharmacological small molecules indicated that TT-10 promoted both cardiomyocyte survival and proliferation (7). Furthermore, when intraperitoneal injections of TT-10 were administered to mice after experimentally induced MI, the treatment promoted cardiomyocyte cell cycle activity and was associated with declines in infarct size. Measures of cardiac function were also significantly better in TT-10–treated animals than in animals administered the delivery vehicle but declined over time in both groups (7).

Here, we focused on the use of PLGA-NPs for TT-10 administration, which is more efficient than conventional systemic delivery and, consequently, could enable patients to be treated with lower drug doses, thereby reducing the risk of treatment-related adverse events as well as the demand for large-scale TT-10 manufacturing protocols. The beneficial effects associated with free (unencapsulated) TT-10 administration in Hara et al. (7) were achieved with a dose of approximately 250 μg/mouse (i.e., 10 mg/kg × ~25 g/mouse), which is more than 3700-fold greater than the dose administered to animals in our TT-10–SOL and TT-10–NP treatment groups (67.2 ng). Although it is difficult to compare the local accumulated concentration of TT-10 in myocardium between our TT-10–NPs and Hara’s study, it is expected that an enhanced and a long-lasting effect can be achieved by the TT-10–NP delivery approach based on the prolonged release curve (Figure 3C). This low dose and slow and longer release feature likely contribute to the dramatic increase in efficiency associated with NP-mediated drug administration.

Our results indicated that targeted PLGA-NP–mediated TT-10 delivery improved both the potency and durability of the benefits associated with TT-10 administration: infarct sizes were significantly smaller (by ~20%), and cardiac performance was significantly better, in animals treated with TT-10–NPs than those in the TT-10–SOL, Empty-NP, or DPBS treatment groups. While functional parameters remained stable from weeks 1 to 4 in TT-10–NP animals, measurements in the other 3 groups that underwent MI induction worsened over the same period. TT-10–NP administration was also associated with increases in the frequency of Ki67, PH3, and nuclear Yap expression among cardiomyocytes 1 week after treatment, as well as with declines in cardiomyocyte apoptosis on day 3, which was consistent with observations in cultured hiPSC-CMs and suggests that the benefits of treatment evolved, at least in part, via the Yap/TAZ-induced activation of cell cycle progression and cardioprotective mechanisms. However, proliferating cardiomyocytes were nearly undetectable 4 weeks after TT-10–NP delivery, which may be beneficial, because proliferating cardiomyocytes are typically immature and, consequently, could increase the risk of arrhythmia and disrupt contractile activity. TT-10–NP administration also promoted angiogenesis in the peri-infarct region, which (to our knowledge) has not been previously reported, and Yap/TAZ signaling can be activated by hypoxia in some cancers (14, 15), as well as extracellular matrix proteins (e.g., periostin; ref. 16), growth factors (17, 18), and microRNAs (19, 20), all of which have been linked to cardiomyocyte cell cycle activity during development or under disease conditions. Thus, cardiomyocyte cell cycle activity in mammalian hearts appears to be governed by a broad range of physiological conditions and signaling mechanisms. The cytoprotective effects of TT-10–NP administration likely occurred during the first few days after administration, when cardiomyocyte apoptosis peaks (21), and may have been mediated by declines in the abundance of ROS and ROS-induced DNA damage (7, 21, 22).

Studies of fluorine-containing pharmaceuticals, such as TT-10, are becoming increasingly common, because these pharmaceuticals are metabolically stable (23) and, consequently, have high bioavailability in vivo. However, their efficacy for treatment of cardiovascular disorders is hampered by poor retention at the site of administration. Thus, our results suggest that PLGA NPs could be used to improve the efficiency of treatment administration for numerous cardiovascular drugs (9). Furthermore, although the animals in our current investigation were treated with TT-10–NPs via direct intramyocardial injections during open-chest surgery, PLGA NPs are fully compatible with less invasive clinical delivery methods, such as catheter-based or echo-guided transthoracic myocardial injection.

In summary, the results from our investigation in a mouse MI model demonstrate that measures of cardiac function and infarct size are significantly better when the animals receive intramyocardial injections of TT-10–loaded NPs rather than TT-10–SOL. Thus, PLGA-NP–mediated delivery appears to enhance the potency of TT-10, which may also reduce the occurrence of treatment-related side effects and other safety concerns by enabling the drug to be administered at lower doses. Collectively, these observations support the continued development of TT-10–NPs for the treatment of cardiac disease and provide proof of concept for the PLGA-NP–mediated delivery of other therapeutic agents.

## Methods

### Preparation and characterization of PLGA NPs.

PLGA NPs were prepared via a single-emulsion (oil/water phase) technique as described previously (10). Briefly, a solution of PLGA (100 mg) in dichloromethane (5 mL) with or without TT-10 (300 μL at 2 mg/mL) or coumarin-6 (1 mg) was ultrasonicated at 40% amplitude in 40-second intervals with 20-second pauses for a total of 2 minutes. Then, 20 mL of 1% (w/v) dimethylamine borane-water solution was added, and the mixture was ultrasonicated on ice at 40% amplitude in 40-second intervals with 20-second pauses for a total of 2 minutes. The mixture was slowly transferred into 10 mL 4% (w/v) PVA-water solution, added with 30 mL Milli-Q water (MilliporeSigma) into a 100 mL glass beaker, and stirred for 4 hours until the dichloromethane completely evaporated. Then, the solution was centrifuged at 1000*g* for 10 minutes to remove bulk aggregates, and the supernatant was centrifuged at 45,000*g* for 20 minutes to collect the NPs. The NPs were washed twice by resuspending them in 50 mL Milli-Q water; they were then recollected via centrifugation (45,000*g* for 20 minutes) and frozen at –80°C overnight, lyophilized for 48 hours, and stored at –80°C.

NP size measurements were performed via NP-tracking analysis with a NanoSight NS300 Instrument (NanoSight), and scanning electron microscopy was performed with a Quanta FEG 650 (FEI). For TT-10–loaded NPs, TT-10 release was monitored with a Slide-A-Lyzer MINI Dialysis Device (Thermo Fisher Scientific); the molecular weight cutoff was set at 20,000, and the elution medium consisted of DPBS with 0.1% BSA. Suspensions were incubated at 37°C with constant shaking (300 rpm), and 14.5 mL samples of the elution medium were withdrawn and replaced at the indicated time points. TT-10 concentrations in the collected samples were measured with a UV/Vis spectrophotometer (NanoDrop, Thermo Fisher Scientific); measurements of TT-10 absorbance (λ = ~200 nm) in samples were compared with a curve constructed from known TT-10 concentrations using the Beer-Lambert Law.

### Differentiation of hiPSCs into cardiomyocytes.

The hiPSCs were maintained in 6-well plates with mTeSR medium (Stem Cell Technologies) until 80% confluent and then differentiated into hiPSC-CMs. Briefly, the hiPSCs were cultured in RPMI 1640 medium with 2% B27 minus insulin (RB medium; Gibco), 10 μM CHIR99021 (Stem Cell Technologies), and 1 μg/mL insulin (MilliporeSigma) for 24 hours; in RB medium containing 3 μM CHIR99021 for 48 hours; in RB medium containing 10 μM IWR1 (Stem Cell Technologies) for 48 hours; in RB medium for 48 hours; and then in RPMI 1640 medium supplemented with 2% B27 supplement (Gibco). Differentiated hiPSC-CMs were purified via metabolic selection in glucose-free RPMI 1640 medium (Gibco) containing B27 supplement (Gibco) and 4 mM lactate (MilliporeSigma) for 5 days and then maintained in RPMI 1640 medium supplemented with 2% B27 supplement (Gibco). Experiments were conducted 30 days after differentiation was initiated.

### Cellular uptake of PLGA NPs.

hiPSCs-CMs were seeded onto a Lab-Tek chamber slide system (Nunc, Thermo Fisher Scientific), treated with coumarin-6–loaded NPs (2 μg/mL), incubated at 37°C and 5% CO_2_ for 24 hours; washed twice with DPBS (pH 7.4); and visualized with a fluorescent microscope.

### Mouse MI model.

Eight- to twelve-week-old male and female C57BL/6 mice (The Jackson Laboratory) were anesthetized with inhaled isoflurane (2%), intubated, and ventilated with 2% isoflurane. A left thoracotomy was performed, and the LAD coronary artery was ligated with an 8 to 0 nonabsorbable suture (24). Animals in the TT-10–NP and Empty-NP groups were treated with PLGA NPs that had or had not, respectively, been loaded with TT-10; animals in the TT-10–SOL group were treated with TT-10–SOL; and animals in the DPBS group were treated with DPBS. The total dosage of TT-10 used in both the TT-10–SOL and TT-10–NP groups was 67.2 ng. For the sham group, a suture was positioned around the LAD coronary artery without ligation. Treatments were administered via intramyocardial injection into 3 sites surrounding the infarct; injections were administered with a modified Hamilton needle, and the total volume of the injection was 24 μL (8 μL/site). Intraperitoneal injections of buprenorphine hydrochloride (0.1 mg/kg, Buprenex, Reckitt Benckiser Pharmaceuticals Inc.) and carprofen (5 mg/kg, Rimadyl, Zoetis) were administered after chest closure for pain control.

### Echocardiography.

Animals were lightly anesthetized with 1%–1.5% inhaled isoflurane, and heart rates remained stable at 400–500 bpm. Then, B-mode and 2-dimensional M-mode images were obtained from long-axis and short-axis views with a high-resolution microultrasound system (Vevo 2100, VisualSonics Inc.). Data were analyzed with Vevo analysis software, and LVEF, LVFS, LVEDD, and LVESD were calculated (10).

### Infarct size.

Infarct size was evaluated as described previously (9). Briefly, hearts were excised, fixed in 4% paraformaldehyde (PFA) for 4 hours, immersed overnight in 30% sucrose at 4°C, embedded in OCT compound, and sectioned at a thickness of 10 μm. Every twentieth section from the base to the apex was fixed in Bouin’s solution and stained with Picrosirius Red/Fast Green dyes. Then, images of the stained sections were obtained with an Olympus light microscope and analyzed with NIH ImageJ software. Infarct size was calculated according to the following formula: infarct size = (scar circumferential length × thickness of each of the short axis)/(short-axis LV length × thickness of the short axis) × 100%.

### Immunofluorescence.

For hiPSC-CMs, the cells were immobilized with 4% PFA for 10 minutes; permeabilized with acetone for 1 minute; washed with PBS + 0.1% Tween 20 (PBST); blocked with 10% donkey serum (Life Technologies) for 30 minutes; incubated at 4°C overnight with primary antibodies; and incubated for 40 minutes with fluorophore-linked secondary antibodies; then, nuclei were stained with DAPI at room temperature for 10 minutes. For heart sections, tissues were fixed in ice-cold 4% PFA for 4 hours, immersed in 30% sucrose overnight at 4°C, and cut into 10 μm sections; then, the sections were washed with PBST for 10 minutes, permeabilized with cold acetone for 3 minutes, immobilized with 4% PFA for 10 minutes, blocked with 10% donkey serum for 30 minutes, incubated with primary antibodies at 4 °C overnight, and incubated with fluorophore-linked secondary antibodies for 40 minutes; then, nuclei were stained with DAPI at room temperature for 10 minutes. After staining, analyses were conducted with a fluorescence microscope.

### BrdU labeling.

hiPSC-CMs were incubated with BrdU (10 μmol) in a humidified atmosphere containing 5% CO_2_ at 37°C for 12 hours, fixed with ice-cold 70% ethanol (pH 2.0, 50 mM glycine) for 15 minutes, and then stained with a BrdU Labeling and Detection Kit (Roche Life Science Inc.) as directed by the manufacturer’s instructions.

### TUNEL.

TUNEL staining was performed with an in situ cell death detection kit (Roche). Briefly, cells or heart sections were fixed with 4% PFA at room temperature for 15 minutes, TUNEL-labeled as directed by the manufacturer’s instructions, washed with PBST, blocked with 10% donkey serum (Life Technologies) for 30 minutes, incubated with primary antibodies at 4°C overnight, and incubated with fluorophore-linked secondary antibodies at room temperature for 40 minutes. Then, nuclei were stained with DAPI. Apoptosis was quantified as the proportion of cells that were TUNEL positive.

### Western blot.

hiPSC-CMs were lysed with M-PER Mammalian Protein Extraction Reagent (Thermo Fisher Scientific) supplemented with Halt Protease and Phosphatase Inhibitor Cocktail (Thermo Fisher Scientific). Extracted protein was quantified by BCA protein assay kit (Thermo Fisher Scientific) and denatured by heating at 95°C for 10 minutes. Equal quantity of protein were loaded and separated by 4%–15% precast SDS-PAGE gels (Bio-Rad), and then transferred onto a polyvinylidene difluoride membrane (Bio-Rad). The membranes were blocked with 5% nonfat dry milk and incubated at 4°C overnight with primary antibodies, as described in *Antibodies*. HRP-conjugated secondary antibody was used at 1:4000 dilution for detection. Finally, the membranes were developed with Immobilon Western Chemiluminescent HRP Substrate (MilliporeSigma). Protein signals were imaged with a Bio-Rad ChemiDoc System and analyzed by ImageJ. The housekeeping protein, GAPDH, was used for normalization.

### Antibodies.

The following primary antibodies and second antibodies were used in the study: mouse anti–cardiac troponin T monoclonal antibody (1:100, Thermo Fisher Scientific, catalog MAB1874); rabbit anti–cardiac troponin T monoclonal antibody (1:100, Abcam, catalog ab91605); mouse anti–cardiac troponin T monoclonal antibody (1:100, Thermo Fisher Scientific, catalog MS295P1); goat anti–cardiac troponin I polyclonal antibody (1:100, Abcam, catalog ab188877); rabbit anti-Ki67 monoclonal antibody (1:100, Abcam, catalog ab16667); rabbit anti–phospho-histone H3 (Ser10) polyclonal antibody (1:1000, MilliporeSigma, catalog 06-570); mouse anti–Aurora B monoclonal antibody (1:50, BD Biosciences, catalog 611082); goat anti-human NKX2.5 polyclonal antibody (1:25, R&D Systems, Thermo Fisher Scientific, AF2444); rabbit anti-YAP monoclonal antibody (1:100 for immunofluorescence and 1:1000 for Western blot, Cell Signaling Technology, catalog 14074S); rabbit anti-phospho YAP monoclonal antibody (1:1000, Cell Signaling Technology, catalog 13008S); fluorescein-labeled GSL I IB4 (1:10, Vector laboratories, catalog FL-1201); rabbit anti-α smooth muscle actin polyclonal antibody (1:200, Abcam, catalog ab5694); CyTM 3 donkey anti-rabbit polyclonal antibody (1:200, Jackson ImmunoResearch Laboratory, catalog 711-165-152); Alexa Fluor 488 donkey anti-mouse polyclonal antibody (1:200, Jackson ImmunoResearch Laboratory, catalog 715-545-150); Alexa Fluor 647 donkey anti-goat polyclonal antibody (1:200, Jackson ImmunoResearch Laboratory, catalog 705-605-147); and anti-rabbit HRP linked antibody (1:4000, Cell Signaling Technology, catalog 7074P2).

### Statistics.

All data are presented as mean ± SEM and were evaluated for significance with GraphPad Prism 7 software. Comparisons between 2 groups were conducted with the 2-tailed Student’s *t* test, and comparisons among 3 or more groups were conducted via 1- or 2-way ANOVA with adjustment. *P <* 0.05 was considered statistically significant.

### Study approval.

All protocols were approved by the Institutional Animal Care and Use Committee of the University of Alabama at Birmingham and were performed in accordance with the *Guide for the Care and Use of Laboratory Animals* (National Academies Press, 2011).

## Author contributions

WC and JZ conceived and designed the project. WC and DP performed the experiments and analyzed the data. WC and JZ wrote the manuscript. WC, YZ, JY, YN, and JZ revised the manuscript. JZ supervised the whole project. All the authors approved the submission and publication of the manuscript.

## Supplementary Material

Supplemental data

## Version 1 10/22/2021

Electronic publication
