# Supplementary material for: TT-10–loaded nanoparticles promote cardiomyocyte proliferation and cardiac repair in a mouse model of myocardial infarction
Source: JCI Insight. 2021 Oct 22;6(20):e151987. doi: 10.1172/jci.insight.151987 (PMC8564911; doi:10.1172/jci.insight.151987)
Supplement: Supplemental data [file jciinsight-6-151987-s098.pdf]

Supplemental figures and figure legends

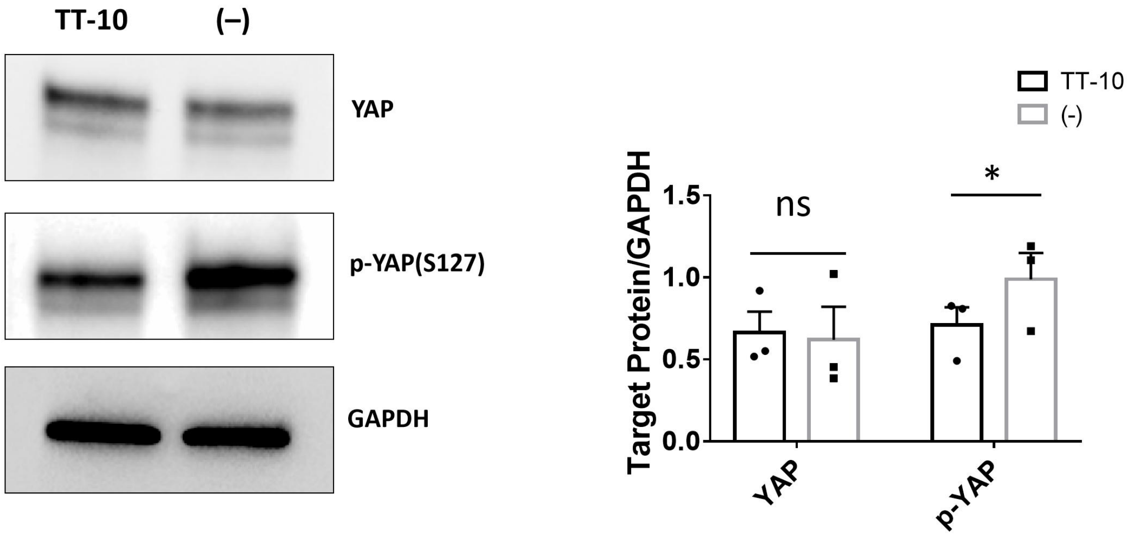

Supplemental Figure 1. TT-10 activated the YAP signaling pathway in cultured hiPSC-CMs. hiPSC-CMs were treated with 10 µM TT-10 for 48 hours, and then YAP and phosphorylated YAP (p-YAP) protein abundance was evaluated via Western blot and quantified via normalization to the abundance of glyceraldehyde phosphate dehydrogenase (GAPDH). $^{*}P<0.05$, Student′s t-test; n = 3 experiments.

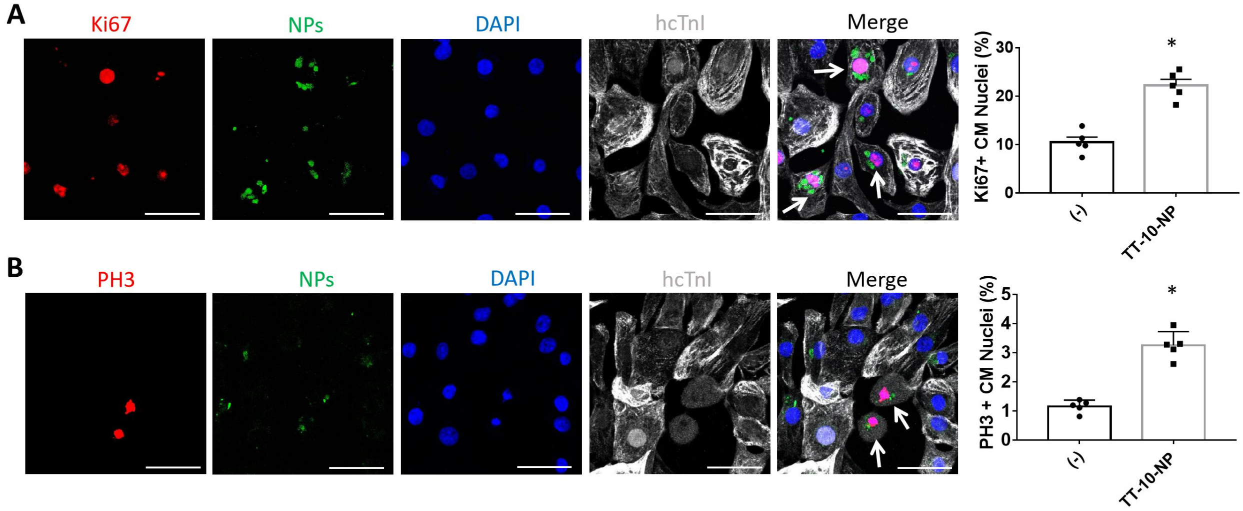

Supplemental Figure 2. TT-10–NPs promoted proliferation in cultured hiPSC-CMs. hiPSC-CMs were cultured for 48 hours with or without 1 mg/mL coumarin-6–loaded TT-10-NPs (green); then, the cells were immunofluorescently stained for the human isoform of cardiac troponin I (hcTnI, gray), and nuclei were identified via DAPI staining (blue). Internalized NPs are identified with an arrow. Scale bar: 20 µm. (A) Proliferation was evaluated via immunofluorescence co-staining for Ki67 and quantified as the percentage of Ki67-positive cells. (B) hiPSC-CMs in the M-phase of the cell cycle were identified via immunofluorescence co-staining for PH3 and quantified as the percentage of PH3-positive cells. *P<0.01, Student's t-test; n = 5 experiments.

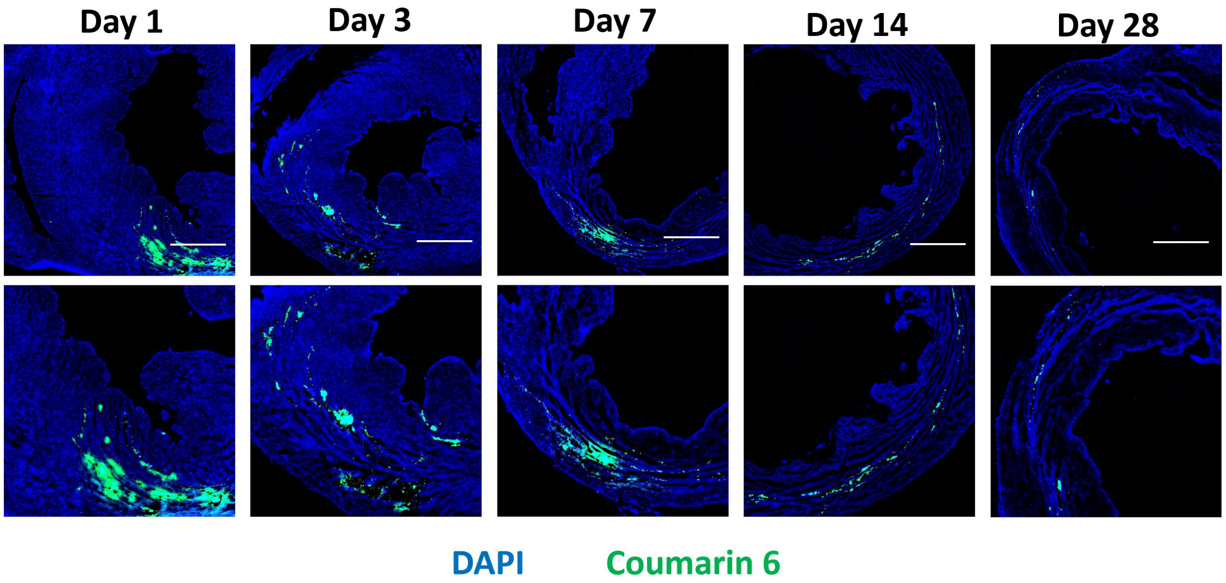

Supplemental Figure 3. TT-10-NPs remain stable after administration to infarcted mouse hearts. NPs were loaded with both TT-10 and coumarin 6 and then intramyocardially injected into infarcted mouse hearts. Animals were sacrificed 1, 3, 7, 14, or 28 days later; then, sections from the BZ were stained with DAPI, and images of coumarin 6 fluorescence were collected. Scale bar: 200 µm (n=2 per time point).
